# Influence of Long-Term Fasting on Blood Redox Status in Humans

**DOI:** 10.3390/antiox9060496

**Published:** 2020-06-06

**Authors:** Françoise Wilhelmi de Toledo, Franziska Grundler, Nikolaos Goutzourelas, Fotios Tekos, Eleni Vassi, Robin Mesnage, Demetrios Kouretas

**Affiliations:** 1 Buchinger Wilhelmi Clinic, 88662 Überlingen, Germany; franziska.grundler@buchinger-wilhelmi.com; 2 Charité–Universitätsmedizin Berlin, Corporate Member of Freie Universität Berlin, Humboldt–Universität zu Berlin and Berlin Institute of Health, 10117 Berlin, Germany; 3 Department of Biochemistry-Biotechnology, School of Health Sciences, University of Thessaly, Viopolis, 41500 Larissa, Greece; nikgkoutz@gmail.com (N.G.); fotis.tekos@gmail.com (F.T.); elenhva.97@outlook.com.gr (E.V.); 4 Gene Expression and Therapy Group, King’s College London, Faculty of Life Sciences & Medicine, Department of Medical and Molecular Genetics, 8th Floor, Tower Wing, Guy’s Hospital, Great Maze Pond, London SE1 9RT, UK; robin.mesnage@kcl.ac.uk

**Keywords:** prolonged fasting, redox biomarkers, antioxidants, GSH, oxidative stress

## Abstract

Fasting is increasingly practiced to improve health and general well-being, as well as for its cytoprotective effects. Changes in blood redox status, linked to the development of a variety of metabolic diseases, have been recently documented during calorie restriction and intermittent fasting, but not with long-term fasting (LF). We investigated some parameters of the blood redox profile in 109 subjects before and after a 10-day fasting period. Fasting resulted in a significant reduction in body weight, improved well-being and had a beneficial modulating effect on blood lipids and glucose regulation. We observed that fasting decreased lipid peroxidation (TBARS) and increased total antioxidant capacity (TAC) in plasma, concomitant with a uric acid elevation, known to be associated with fasting and did not cause gout attacks. Reduced glutathione (GSH), glutathione reductase (GR), glutathione peroxidase (GPx) and catalase in erythrocytes did not show significant changes. In addition, reduction in body weight, waist circumference, and glucose levels were associated to a reduced lipid peroxidation. Similar results were obtained by grouping subjects on the basis of the changes in their GSH levels, showing that a period of 10 days fasting improves blood redox status regardless of GSH status in the blood.

## 1. Introduction

All living organisms alternate between periods of food intake and fasting throughout their lives. Humans fast daily during night sleep phases, which generally last from 6 to 10 h. Longer periods of fasting were quite frequent in the course of evolution due to seasonal cycles. Food access could also be difficult and conservation technologies were limited. Humans and animals were obliged to survive without exogenous food until it was available again, thus switching their metabolism toward endogenous fat utilization [1,2,3]. The first publications of long-term fasting (LF) came up in the 20th century [4].

LF, also called sometimes periodic fasting [5,6], refers to total abstinence or a strong reduction in energy intake for from 2 to 21 days or more [7]. It has been extensively studied in free-living animals which survive on body energy stores, such as in migrating birds, hibernating animals and penguins [8,9]. LF in humans has been practiced for decades under medical supervision in specialized centers [10]. LF has been used to treat several pathologies [7]. In contrast to LF, intermittent fasting (IF) is defined as periods of fasting lasting from 12 to 48 h, and is the most studied fasting regimen [11,12,13]. It appears that IF increases life- and health-span through weight and fat loss but also through a reduction in reactive oxygen species (ROS) production, improved glucose regulation and lipid metabolism, increased cellular stress resistance and decreased inflammation [3,12]. ROS were found to modulate the redox status by activating transcription factors and apoptosis [14].

In general, 10–16 h after the last food intake, blood sugar, insulin and IGF-1 levels decrease, while glucagon and growth hormone increase, orchestrating the fasting metabolism. Ketone bodies are used as energy sources and regulate the expression and activity of various transcription factors. These metabolic changes are known as “metabolic switch” from liver-derived glucose to adipose-cell-derived ketones (G-to-K) and the reverse pathway K-to-G upon refeeding [15,16]. During fasting circulating amino acids decrease, which induces the deactivation of the nutrient-dependent signaling pathway mammalian target of rapamycin (mTOR), leading to reduced cell growth, an enhanced autophagy and a decreased activation of the nuclear factor kappa B (NF-κB) pathway, thus reducing inflammatory processes. On the one hand, the decrease in the ATP/AMP ratio will activate AMP-activated protein kinase (AMPK). On the other hand, it will induce a mild oxidative eustress [17]. Both lead to the stimulation of antioxidant and cytoprotective enzymes synthesis such as superoxide dismutase, catalase, peroxidase, glutathion *S*-transferase. Several transcription factors like sirtuins (SIRT) play a pivotal role in mitochondrial biogenesis, such as a fork head box O (FoxO) that activates cellular autophagy and nuclear factor erythroid-2-related factor 2 (Nrf2). Nrf2 activates several key genes for the production of antioxidants, such as glutathione and specific antioxidant enzymes [18,19,20,21,22,23,24].

There issome evidence that redox mechanisms are related to the beneficial properties of LF, IF and CR, in the same way that exercise seems to be related to redox mechanisms [25,26]. The G-to-K switch leads to an increased number of mitochondria (mitohormesis) in order to accelerate aerobic catabolism of fats through the mechanisms of AMPK and SIRT [27,28,29]. Semi-quantitative metabolomic analysis in human whole-blood plasma and red blood cells during 34–58 h fasting in four subjects showed that 44 of ~130 metabolites increased rapidly, including antioxidant molecules. However, the small number of subjects and the relatively short period of fasting limited the conclusiveness of this study. Therefore, in our prospective study, we examined the potential ability of a 10-day fasting period of the peer-reviewed Buchinger Wilhelmi fasting program [10] to influence redox status in the blood plasma and erythrocytes of 109 subjects. For this purpose, specific redox biomarkers with a high translational potency were evaluated: TAC, TBARS, GSH, GR, GPx and Catalase [30].

## 2. Materials and Methods 

### 2.1. Ethics Statement

This interventional study was conducted in accordance with the principles of the Declaration of Helsinki and was approved by the medical council of Baden-Württemberg, Stuttgart, under the application number F-2018-118 on 12 February 2019. The trial was registered on 20 February 2019 in the German Clinical Trials Register (DRKS-ID: DRKS00016657). Written informed consent was obtained from all participants prior to enrolling into the study. The recruitment was conducted between 15 September 2019 and 18 November 2019, and the follow-up was completed till 2 January 2020. All participants were in general good health. Two time-points were specified for each individual. The baseline examination was conducted before starting of the fast (time point #1, pre) and the second examination was done at the 10th ± 3 fasting day (time-point #2, post).

### 2.2. Participants

The participants were recruited among the subjects who were admitted to the Buchinger Wilhelmi Clinic (BWC), and their age was between 18 and 70 years. Only subjects who underwent at least 7 days of fasting and conducted two laboratory examinations at the beginning and at the end of the experiment (10 ± 3 days) were included. The intake of micronutrient supplements was advised to be stopped one week before and during the fasting period, with the exception of magnesium supplementation. Subjects with chronic psychiatric diseases were excluded, as well as pregnant or lactating women, according to the guidelines of fasting therapy [10]. Moreover, subjects that were not able to communicate in German, English or French, and thus were unable to answer the questionnaires, or subjects that were participating in another study, were also excluded. Out of the 182 subjects who were admitted to the BWC, 37 did not meet the inclusion criteria, 35 subjects did not want to participate and one subject terminated the study prematurely due to low hemoglobin and sodium levels. A total of 109 subjects completed the study.

### 2.3. The Fasting Protocol

A day before the first day of fasting, the subjects received a 600 kcal diet of either rice and vegetables or fruits. On the first day of fasting, a laxative (20–40 g Na_2_SO_4_ in 500 mL water) was administered. Every other day during fasting, an enema was applied to remove intestinal remnants and desquamated mucosal cells. A daily calorie intake of around 250 kcal was available daily in form of 20 g honey, as well as 250 mL freshly squeezed organic juice at midday and 250 mL of vegetable soup in the evening. The subjects were advised to drink at least 2–3 L of water and herbal teas per day. The fasting period was followed by a stepwise reintroduction of an ovo-lacto-vegetarian organic diet 800 to 1600 kcal/day. The fasting program followed the guidelines of the fasting therapy [10]. Specialized physicians and trained nurses supervised the course of the program, that also included physical exercise and other individual treatments [5].

### 2.4. Clinical Data

All measurements were performed at the first and at the last day of fasting (10 ± 3 fasting day) in the morning in the fasted state. Before the start, the subjects underwent a thorough physical examination. The height of the subjects was measured with seca 285 (Seca, Hamburg, Germany) and the waist circumference was assessed with a measuring tape halfway between the lowest rib and the iliac crest. Body weight was measured daily (Seca 704/635, Seca, Hamburg, Germany) between 7:00 a.m. and 9:00 a.m. by trained nurses according to a standardized protocol, while subjects wore light clothing and no shoes. Possible adverse events had to be reported by the medical staff. Emotional (EWB) and physical well-being (PWB) was daily self-rated on numeric scales from 0 (very bad) to 10 (excellent) under nurses’ supervision.

### 2.5. Blood Collection and Handling for the Measurement of Redox Biomarkers

Blood samples were drawn at baseline in the first morning after arrival between 7.30 and 9.00 a.m., and at the 10 ± 3 fasting day by trained medical-technical assistants. Blood samples (10 mL) were drawn from a forearm vein with subjects in a seated position. Blood was collected in ethylenediaminetetraacetic acid (EDTA) tubes, centrifuged immediately (1370× *g*, 10 min, 4 °C), and the plasma was collected and used for the measurement of total antioxidant capacity (TAC) as a crude biomarker of the antioxidant potential of plasma and thiobarbituric acid reactive substances (TBARS) concentration as a biomarker of lipid peroxidation. The remaining packed erythrocytes were lysed with dH_2_O (1:1 *v*/*v*), inverted vigorously, centrifuged (4020× *g*, 15 min, 4 °C) and the erythrocyte lysate was collected for the measurement of Catalase, Glutathione Reductase (GR) and Glutathione Peroxidase (GPx) activity. A portion of erythrocyte lysate (500 μL) was treated with 5% trichloroacetic acid (TCA) (1:1 *v*/*v*), vortexed vigorously and centrifuged (28,000× *g*, 5 min, 4 °C). The supernatant was removed, treated again with 5% TCA (1.3:1 *v*/*v*) and centrifuged (28,000× *g*, 5 min, 4 °C). The clear supernatant was transferred to plastic tubes and was used for the determination of GSH concentration. A blood aliquot (1 mL) was immediately mixed with EDTA to prevent clotting for hematology measurements. Plasma and erythrocyte lysate samples were then stored at −80 °C until the biochemical analyses were performed. Routine laboratory blood parameters were performed in the laboratory MVZ Labor Ravensburg. The liver enzymes (serum glutamic oxaloacetic transaminase (GOT), serum glutamate pyruvate transaminase (GPT), serum gamma-glutamyl transferase (GGT), alkaline phosphatase (AP)), kidney parameters(glomerular filtration rate (GFR), urea, creatinine, uric acid), total cholesterol and triglycerides, as well as glucose, were analyzed with ADVIA 2400 (Siemens Health care GmbH, Erlangen, Germany).

### 2.6. Protocols for the Measurement of Redox Biomarkers

GSH levels were measured according to Reddy et al. [24] as previously described [31]. Twenty microliters of erythrocyte lysate treated with 5% TCA were mixed with 660 μL of 67 mM sodium potassium phosphate (pH = 8) and 330 μL of 1 mM 5,5-dithiobis-2 nitrobenzoate (DTNB). The samples were incubated in the dark at room temperature (RT) for 15 min and the absorbance was monitored at 412 nm. GSH concentration was calculated on the basis of the molar extinction coefficient of DTNB (13.6 L/mmol/cm). The intra- and inter-assay CVs for GSH were 3.1% and 4.5%, respectively.

Catalase activity was determined using the method of Aebi [32] as previously described [31]. Briefly, 4 μL of erythrocyte lysate (diluted 1:10) was added to 2991 μL of 67 mM sodium potassium phosphate (pH = 7.4) and the samples were incubated at 37 °C for 10 min. Five microliters of 30% hydrogen peroxide (H_2_O_2_) was added to the samples and the change in absorbance was immediately monitored at 240 nm for 130 s. Calculation of the catalase activity was based on the molar extinction coefficient of H_2_O_2_ (40 L/mol/cm). The intra- and inter-assay CVs for catalase were 6.2% and 10.0%, respectively.

The determination of TAC was based on the method of Janaszewska and Bartosz [33]. Briefly, 20 μL of plasma was added to 480 μL of 10 mM sodium potassium phosphate (pH = 7.4) and 500 μL of 0.1 mM 2,2-diphenyl-1-picrylhydrazyl (DPPH^•^) free radical, and the samples were incubated in the dark for 30 min at RT. The samples were then centrifuged (20,000× *g*, 3 min, 20 °C) and the absorbance was monitored at 520 nm. TAC is presented as mmol of DPPH^•^ reduced to 2,2-diphenyl-1-picrylhydrazine (DPPH:H) by the antioxidants of plasma. The intra- and inter-assay CVs for TAC were 2.9% and 5.4%, respectively.

For TBARS determination, a slightly modified assay of Keles et al. was used [34]. According to this protocol, 100 μL of plasma was mixed with 500 μL of 35% TCA and 500 μL of tris-hydroxy-methyl-aminomethane hydrochloride (Tris–HCl) (200 mM, pH = 7.4) and incubated for 10 min at RT. One milliliter of 2 M sodium sulfate (Na_2_SO_4_) and 55 mM thiobarbituric acid (TBA) solution was added and the samples were incubated at 95 °C for 45 min. The samples were cooled on ice for 5 min and vortexed after adding 1 mL of 70% TCA. The samples were centrifuged (15,000× *g*, 3 min, 20 °C) and the absorbance of the supernatant was monitored at 530 nm. The calculation of TBARS concentration was based on the molar extinction coefficient of malondialdehyde (156,000 (L/mol/cm). The intra- and inter-assay coefficients of variation (CV) for TBARS were 3.9% and 5.9%, respectively.

Glutathione Peroxidase (GPx) activity was measured according to Flohe and Gunzler, as previously described by Veskoukis et al. [31]. Briefly, 100 μL of GR (0.24 U), 100 μL of 10 mM GSH and 100 μL of RBCL (diluted 1/100) were added to 500 μL of phosphate buffer (100 mM, pH 7) and the mixture was incubated for 10 min at RT. Then, 100 μL of NADPH (1.5 mM) in 0.1% NaHCO_3_ solution was added in a cuvette and was incubated for 3 min at RT. The reaction was started by adding 100 μL of *t*-BuOOH (12 mM) and the decrease in absorbance at 340 nm was monitored for 5 min. The non-enzymatic reaction rate was assessed by replacing the enzyme sample by buffer. The calculation of GPx activity is based on the molar extinction coefficient of NADPH (6200 L/mol/cm).

Glutathione reductase (GR) activity was measured according to a protocol from Tietze et al., which was modified by Smith et al. and described previously by Veskoukis et al. [31]. Specifically, the reaction mixture of the sample contains 700 μL of phosphate buffer (200 mM, 1 mM EDTA, pH 7.5), 250 μL of DTNB (3 mM) dissolved in phosphate buffer (200 mM), 50 μL of β-NADPH (2 mM) also dissolved in phosphate buffer (200 mM) and 50 μL of GSSG (20 mM) dissolved in phosphate buffer (200 mM). The reaction started with the addition of 25 μL of RBCL (diluted 1/20) and the change in absorbance was monitored at 412 nm for 1 min. A mixture containing 25 μL of GR (1 U/mL) instead of RBCL was used as standard. The GR activity was assessed based on the absorbance change in the standard sample.

### 2.7. Statistical Analysis

The statistical analysis was performed using R version 3.6.1. Pair-wise comparisons were performed using a paired *t*-test. Differences at baseline were evaluated using an ANOVA test (continuous variables) or a chi-square goodness of fit test (categorical variables) using the R package ‘arsena’. We also evaluated associations between the different clinical parameters using linear mixed models (R package lmerTest), considering the time-point as a covariate and the repeated measure (patient grouping) as a random effect. Data were visualized using the R package ggplot2. Data are presented as mean ± SD.

## 3. Results

The cohort was middle-aged, moderately active and predominantly female (Table 1). LF resulted in a significant reduction in body weight by 4.5 ± 1.9 kg. In addition, a significant decrease in waist circumference by 5.3 ± 2.9 cm was observed (Table 2). The intervention was well-tolerated. Patient emotional (EWB) and physical well-being (PWB), measured on numeric scales from 0 (very bad) to 10 (excellent), increased (+1.6 PWB, +1.3 EWB). Only one 53-year old woman had to interrupt her fast on the 7th day due to general weakness and mild hyponatremia (135 mmol/L).

Glucose (14.2%), triglycerides (26.2%) and cholesterol (11.8%) decreased significantly during LF (Table 2). The liver enzymes GPT (31.5%) and GOT (58.8%) were increased on the 10th fasting day, whereas AP (3.45%) and GGT (30.2%) decreased. The kidney parameters GFR (2.8%) and urea (30.2%) were reduced, and creatinine (3.8%) as well as uric acid (48.7%) increased.

**Figure 1:**
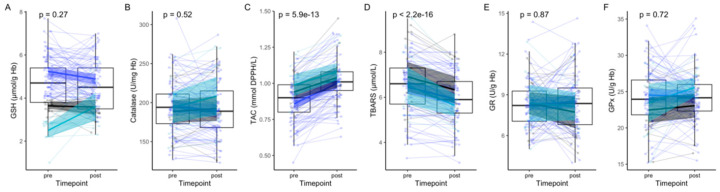
The effects of the 10-day fast on the mean levels of redox biomarkers in a group of 109 subjects. There was no change in GSH levels (**A**). We highlighted 3 subgroups based on the variations in their GSH levels (increase, light blue; decrease, dark blue; unchanged, black) to understand if baseline GSH levels could influence the response to fasting. Catalase (**B**) levels showed no changes either, while TAC (**C**) was significantly increased, and TBARS (**D**) levels were significantly decreased. GR (**E**) as well as GPx (**F**) levels were unchanged.

A previous study suggested that, in subjects with metabolic syndrome and type 2 diabetes, differences in the redox status could be related to GSH levels that differ between high or low GSH values [35]. We then hypothesized that initial levels of GSH could predict the evolution of this parameter, as well as the influence of the effect of fasting. In order to test this hypothesis, three groups of subjects were formed based on clinically relevant GSH baseline values: the first one with low GSH levels (GSH <3 µmol/g Hb), the second one with high GSH levels (GSH >4 µmol/g Hb), and the third group with intermediate GSH values (between 3.1 and 3.9 µmol/g Hb) (Figure 1). Interestingly, subjects who had high GSH concentrations also had low urea values (Table 1). In addition, subjects with low GSH values presented the highest glucose values (Table 1). However, most of the parameters did not different between the GSH groups (Table 1). 

Changes in redox status were then evaluated. There was no change in GSH (Figure 1A) or catalase (Figure 1B) levels after 10 days of fasting. TAC was significantly increased by 12.7% (Figure 1C), with a strong correlation with uric acid levels, and TBARS concentration was decreased by 9.6% (Figure 1D) compared to baseline. The statistical analysis was repeated by considering the GSH classification in three subgroups as a covariate. All three subgroups showed the same results as the whole group regarding TBARS and TAC (Figure 1C,D). GSH levels tend to decrease in the high GSH group, and increase in the low GSH group. However, the homogeneity of variances was not different after a Bartlett’s test, which indicates that this trend could be artificial. 

**Figure 2:**
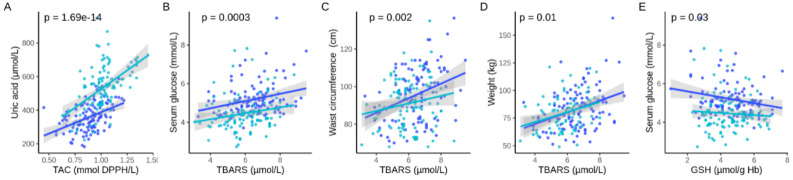
Changes in redox parameters during fasting were associated with changes in clinical parameters. We evaluated associations between the four redox parameters measured in this study (GSH, Catalase, TBARS, TAC) with weight, waist circumference, glucose and uric acid serum levels. The 5 significant associations (**A**–**E**) are presented in this figure. Dot plots shows the correlations between the different parameters for pre-fasting (dark blue) and post-fasting (light blue) levels.

**Figure 3:**
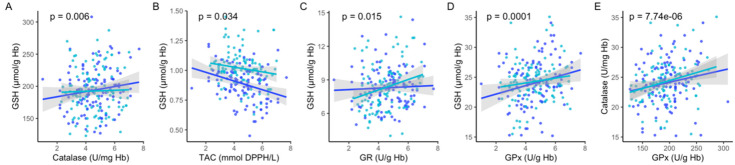
Associations between the different redox parameters measured in this study. We evaluated associations between the six redox parameters measured in this study (GSH, Catalase, TBARS, TAC, GR, GPx). The 5 significant associations (**A**–**E**) are presented in this figure. Dot plots show the correlations between the different parameters for pre-fasting (dark blue) and post-fasting (light blue) levels.

We ultimately evaluated if the changes in redox status can correlate with changes in clinical parameters such as weight loss, or biochemical markers of liver and kidney function. We first analyzed if pre and post fasting differences were significant for the different parameters. Most parameters changed significantly (Table 2), which confirmed observations for a cohort of 1422 subjects in our previous study with other participants [5]. Then, we used linear-mixed model to test if the levels of the redox parameters are associated with changes in clinical parameters. This predictive ability was not found in parameters like AP, GPT, GOT, creatinine, triglycerides, cholesterol, and GGT. Our analysis found that TAC levels were strongly associated to uric acid levels (*p* = 1.69 × 10^−14^) (Figure 2A). There was a significant association between TBARS and glucose serum levels (*p* = 0.0003) (Figure 2B), waist circumference (*p* = 0.002) (Figure 2C), and body weight (*p* = 0.01) (Figure 2D). This result suggests that the production of TBARS could be predicted by variations in glucose levels. It also suggests that the reduction in glucose caused by fasting is associated with reduced production of TBARS. It also suggests that glucose levels could be associated with GSH levels (*p* = 0.03) (Figure 2E). Given the known roles of GR and GPx in GSH metabolism, it is perhaps not surprising to observe that GSH levels were associated to GR (*p* = 0.02) and GPx (*p* = 0.0001) levels (Figure 3).

**Figure 4:**
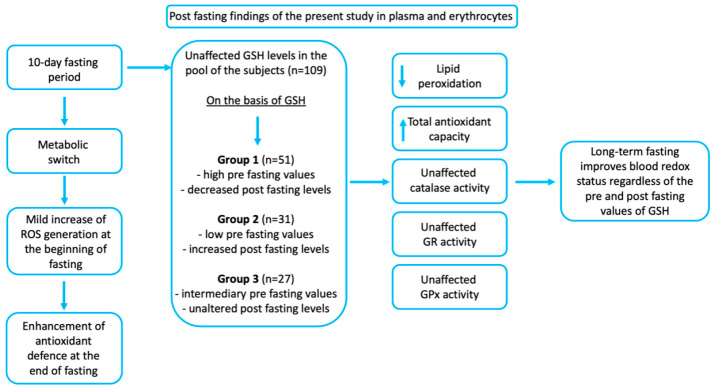
The beneficial effects of long-term fasting and the findings of the present study. It is established from the literature that fasting leads to a metabolic switch, to decreased reactive oxygen species (ROS) generation at the beginning of fasting and to enhancement of antioxidant defense mechanisms at the end of fasting. This could lead to an increase in health- and life-span and general well-being. In the present study, a 10-day fasting protocol decreased lipid peroxidation and increased total antioxidant capacity in plasma in a pool of 109 participants. Fasting improved the blood redox status of participants in the 3 GSH subgroups regardless the baseline GSH concentration.

Overall, our study suggests that a 10-day fast caused changes in blood redox status, which can be linked to improvements in some clinical parameters (Figure 4).

## 4. Discussion

Fasting has gained popularity worldwide in the last decade. We documented the changes in redox status during a 10-day fast and their associations with markers of metabolic health. Fasting reduced plasma lipid peroxidation, as documented by the decrease in TBARS concentration. It increased TAC in plasma, reflecting an enhancement of the antioxidant mechanisms. In addition, we also analyzed the role of GSH baseline values on the effects of fasting. This could be an important issue since the therapeutic efficiency of any redox-altering intervention depends on the baseline levels of endogenous antioxidants, also called antioxidant status [36].

Fasting represents an important physiological stimulus to the human body, with the metabolic switch from G-to-K triggering a wide range of reactions. Various metabolic changes are induced at cellular and metabolic levels as well as at the level of gene expression in genes associated with aging and diminished oxidative stress [37,38,39,40,41]. The deactivation of mTOR, an important signaling pathway involved in the regulation of redox status, has been shown to extend the lifespan of various organisms and to activate FoxO proteins that regulate autophagy and mitophagy, which triggers the recycling of cell elements [20,42,43,44,45,46,47]. In addition to autophagy activation, fasting and calorie restriction inhibit nuclear transcription factor-kB (NF-kB), the main regulator of inflammation, exerting anti-inflammatory activity [48,49].

In this study, biochemical parameters related to the function of the cardiovascular system, liver and kidneys were also examined. Fasting caused a significant reduction in weight of 6.1%, abdominal circumference of 5% and a decrease in systolic and diastolic blood pressure, glucose, triglyceride and cholesterol levels which are risk factors for cardiovascular diseases. Previous studies have shown a similar effect of fasting on these biomarkers [5,50]. Additionally, an increase in physical and emotional well-being and a lack of hunger in most subjects also documented the good tolerability of LF in a recent study on 1422 subjects [5]. In terms of liver function, we studied four biomarkers: GOT and GPT showed a discrete but significant increase. At the same time, GGT decreased rapidly. These findings have been repeatedly published and, when the fasting period is long enough, a return of GOT and GPT to or under baseline was documented [5]. The well-being, physically and emotionally, increased. Together with the decrease in GGT, this situation seems different to the usual picture of liver damage. As for the indicators of renal function after fasting, there was a small but significant decrease in GFR, as well as in urea levels. In addition, there was an increase in creatinine and uric acid levels. These changes, except for uric acid, were within the norm. A transitory elevation in uric acid has been observed in numerous studies of different fasting lengths and is considered as one of the fasting markers [26]. The increase in uric acid concentrations that exceeds the norm range, is not linked, neither in our study nor in a previous study, to gout attacks or renal dysfunction [5]. The increased concentration of blood uric acid is related on the one hand to an initial increase in purine catabolism, and on the other hand to retention caused by competitive tubular renal secretion with ketone bodies. The latter are preferentially secreted in urine during fasting [51].

We have hypothesized that the beneficial effects of fasting could be related to the human redox status. We found that lipid peroxidation measured was decreased while TAC was increased significantly post-fasting compared to the pre-fasting values. This suggested that fasting acts protectively by activating the antioxidant machinery. A decrease in TBARS has also been observed in a study examining the effect of intermittent fasting in a Muslim cohort [52]. In addition, we observed a correlation between baseline TBARS values and weight. The association between obesity and increased lipid peroxidation has been documented earlier [53]. This was also observed in the context of Ramadan intermittent fasting [54]. We found a positive correlation between TBARS and glucose levels, and a negative correlation between GSH and glucose. The patients with higher glucose levels had higher lipid peroxidation and lower GSH levels. This is probably due to mechanisms by which the increased glucose leads to ROS generation through NADPH oxidases up-regulation and glucose oxidase [55,56,57]. The observed increase in TAC is strongly associated with an increase in plasma uric acid, as it has been found that uric acid is a potent plasmatic antioxidant [5,26,53,58]. A study in asthmatic individuals showed decreased levels of oxidative stress in circulation, e.g., protein carbonyls, nitrotyrosine, 8-isoprostane levels that correlated with an increase in uric acid [59]. 

Regarding the four other biomarkers, no significant changes were observed in the mean pre/post values of Catalase, GPx, GR activity or in GSH levels. They were associated, which we expected, given the known regulating role of GPx and GR and GSH levels. The latter has also been reported in a very small group of four subjects by means of metabolomics [26]. Our results indicate that there was no effect on GPx and GR activity, and consequently the GSH redox cycle was not significantly affected after 10 days.

Since grouping subjects on the basis of the changes in their GSH levels was suggested to provide insight into the development of metabolic diseases in previous studies [35], we divided the subjects in three subgroups: in group 1 (46.8% of the subjects), high GSH concentrations decreased, in group 2 (28.4% of the subjects), low GSH concentration increased, possibly due to enhanced biosynthesis [60], and group 3 (24.8% of the subjects), in which GSH concentration remained unchanged. We hypothesized that fasting would normalize GSH concentrations, leading to a more homogenous variance at the end of the intervention, but the present data do not allow us to draw a clear conclusion. Further studies should be performed with daily measurements on groups of patients with abnormal GSH values, such as individuals with chronic diseases (e.g., cancers or neurodegenerative diseases) or harboring mutations of the gene MTHFR, which has important role in cystein metabolism.

Clustering the subjects according to their baseline (i.e., pre-fasting) GSH values could have a predictive potential for LF outcomes. GSH concentration has been shown to correlate with different metabolic diseases [35]. Low GSH levels have been associated with oxidative stress while high GSH values probably indicate reductive stress, two conditions that may be involved in different metabolic disorders [23,61,62]. The issues of individual baseline reductive and oxidative stress in terms of individuality has been previously examined and has led to the conclusion that antioxidant treatments aiming to improve redox profile are dependent on the baseline values of intrinsic antioxidants. Specifically, it has been demonstrated that vitamin C and E administration decreases oxidative stress only when the levels of the biomarkers of macromolecule oxidative modification are high [63], whereas acute exercise induces oxidative stress in unprotected individuals with low antioxidant values at rest [64].

Interestingly, TAC was increased in the group as a whole, as well as in each of the subgroups. Similarly, TBARS and Catalase, GPx and GR activity variations were consistent in the whole group as well as in the subgroups (Figure 2).

The fuel switch from glucose to fat at the onset of fasting increases moderately the production of ROS and induces mild oxidative stress, sometimes called eustress [17]. This has signaling activity and triggers a useful adaptive response that modulate antioxidant defense and increases the ability of cells to cope with oxidative stress [65]. Calorie restriction activates Nrf2, which is a “cellular regulator of oxidative factors” that affects ROS homeostasis by regulating the antioxidant defense systems through regulations of gene expression of several target genes [27,66]. These include the induction of a superoxide dismutase, peroxiredoxin and glutathione peroxidase, crucial antioxidant enzymes [67]. We therefore assume that the regulation of antioxidant mechanisms is related to their levels before fasting. It was pointed out that various metabolic diseases such as diabetes and cancer are related to oxidative redox potential [68]. There are subjects with oxidative stress but also subjects with reductive stress [55]. An excessively reducing environment may be responsible for the improper folding of proteins in the endoplasmic reticulum [55,56,57]. In the reducing environment, disulfide bonds between cysteine residues cannot develop, and ultimately proteins do not acquire their normal tertiary structure, which in the long run may lead to loss of function, resulting in the onset of disease [57,69]. We could postulate that individuals with a high pre-fasting GSH concentration (group 1) are in a more intense reductive cellular environment, whereas the subjects with low levels of pre-fasting GSH levels (group 2) need to enhance the protective GSH levels. In group 3, characterized by intermediate baseline GSH levels, it can be postulated that the absence of change was due to the adequate redox status pre-fasting. A more detailed analysis of daily changes in redox status could provide more insight into this hypothesis. We cannot exclude that the cut-off at ten days does not reflect the changes in all subjects. Furthermore, the reductive stress induced by exercise, although generally known to be beneficial, can be detrimental for individuals with low GSH values, but not for those individuals with increased baseline GSH values [36]. The effects of redox-altering stimuli, such as antioxidant administration and exercise, on blood redox status, are highly dependent on the baseline values of the intrinsic antioxidant mechanisms. However, our study shows that this is not the case for a period of 10 days fast, as presently studied, since its effects on redox biomarkers are the same regardless of the GSH baseline values. Other studies that analyzed antioxidant enzymes such as catalase and were performed on animals have observed an increase after prolonged fasting [54].

## 5. Conclusions

In conclusion, this study reports that a 10-day fasting protocol leads to weight loss, improved cardiovascular parameters, decreased lipid peroxidation and increased antioxidant capacity of plasma in 109 subjects. We proposed to discriminate the subjects in three groups on the basis of their baseline (i.e., pre-fasting) GSH values and found that the impact of this fasting protocol on the lipid peroxidation and total antioxidant capacity was the same in all three groups. This is one of the first studies to describe the effects of LF on blood redox status in humans and to show that these effects are independent from the pre-fasting values of GSH. Further studies with daily measurements are needed to understand better the evolution of the redox status in LF.

## Figures and Tables

**Table 1:** Baseline characteristics in all, as well as in the 3 subgroups, classified according to glutathione (GSH) level variations during fasting. We evaluated the whole group of subjects and evaluated if the 3 subgroups differed from the whole groups in their baseline parameters (* *p* < 0.05; ** *p* < 0.01; *** *p* < 0.001).

Parameters	All (*n* = 109)	High GSH (*n* = 77)	Intermediate (*n* = 18)	Low GSH (*n* = 14)
Age, years	57.0 (±10.5)	56.4 (±10.4)	56.2 (±11.0)	60.5 (±9.8)
Female, n	68 (62%)	52 (68%)	10 (56%)	6 (43%)
Male, n	41 (38%)	25 (32%)	8 (44%)	8 (57%)
Waist circumference, cm	95.8 (±14.2)	95.1 (±14.8)	94.1 (±13.2)	102.5 (±10.4)
BMI, kg/m^2^	28.34 (±6.01)	28.39 (±6.33)	27.63 (±5.88)	29.07 (±4.29)
Weight, kg	82.9 (±18.8)	81.6 (±19.0)	84.3 (±21.3)	88.6 (±13.4)
Physical activity, h/week	4.6 (±4.6)	4.7 (±4.7)	4.1 (±4.6)	4.8 (±3.7)
Emotional well-being	6.4 (±1.9)	6.3 (±1.9)	6.2 (±1.8)	7.3 (±2.0)
Physical well-being	6.1 (±1.8)	6.0 (±1.8)	6.1 (±1.7)	6.8 (±2.2)
Systolic BP, mmHg	131.9 (±19.3)	133.6 (±18.5)	125.5 (±18.3)	130.9 (±25.0)
Diastolic BP, mmHg	83.8 (±10.4)	84.3 (±10.3)	80.8 (±12.5)	84.8 (±8.1)
Glucose, mmol/L	5.20 (±0.86) *	5.13 (±0.74)	5.05 (±0.59)	5.78 (±1.41)
Triglycerides, mmol/L	1.45 (±0.66)	1.45 (±0.69)	1.39 (±0.58)	1.58 (±0.65)
Cholesterol, mmol/L	5.77 (±1.16)	5.82 (±1.16)	5.47 (±1.03)	5.85 (±1.29)
AP, µkat/L	1.16 (±0.33)	1.17 (±0.32)	1.02 (±0.31)	1.25 (±0.39)
GPT, µkat/L	0.54 (±0.35)	0.54 (±0.37)	0.48 (±0.21)	0.63 (±0.40)
GOT, µkat/L	0.34 (±0.14)	0.33 (±0.15)	0.33 (±0.09)	0.38 (±0.17)
GGT, µkat/L	0.63 (±0.68)	0.66 (±0.74)	0.54 (±0.56)	0.57 (±0.46)
GFR, mL/min/1.73 m^2^	92.8 (±13.7)	93.7 (±12.5)	92.3 (±18.7)	88.2 (±14.2)
Creatinine, µmol/L	69.09 (±13.47)	67.36 (±11.51)	72.02 (±16.29)	75.08 (±18.06)
Urea, mmol/L	4.731 (±1.222) *	4.548 (±1.236)	5.00 (±1.18)	5.41 (±0.93)
Uric acid, µmol/L	358.9 (±83.64)	348.7 (±78.39)	368.5 (±111.7)	403.8 (±58.18)
GSH, µmol/g Hb	4.63 (±1.3) ***	5.2 (±0.9)	3.64 (±0.3)	2.50 (±0.52)
Catalase, U/mg Hb	194.2 (±33.6)	195.6 (±35.1)	188.4 (±28.3)	193.8 (±32.9)
TAC, mmol DPPH/L	0.88 (±0.1) **	0.85 (±0.2)	0.90 (±0.1)	0.98 (±0.1)
TBARS, µmol/L	6.5 (±1.3)	6.4 (±1.2)	6.9 (±1.3)	6.5 (±1.4)
GR, U/g Hb	8.3 (±1.8)	8.3 (±1.8)	8.4 (±1.6)	8.4 (±2.1)
GPx, U/g Hb	24.1 (±3.9)	24.6 (±3.9)	21.3 (±6.0)	23.5 (±4.2)

BMI, body-mass-index; BP, blood pressure; AP, alkaline phosphatase; GOT, serum glutamicoxaloacetictransaminase; GPT, serum glutamatepyruvatetransaminase; GGT, serum gamma-glutamyl transferase; GFR, glomerular filtration rate; GR, glutathione reductase; GPx, glutathione peroxidase.

**Table 2:** Changes in clinical parameters observed in all subjects. Statistical differences between the repeated-measures comparison was determined using a paired *t*-test.

Parameters	Pre-Fasting	Post-Fasting	*p*-Value
Waist circumference, cm	95.8 (±14.2)	91.0 (±13.1)	<2 × 10^−16^
BMI, kg/m^2^	28.34 (±6.01)	27.68 (±5.20)	<2 × 10^−16^
Weight, kg	82.9 (±18.8)	79.9 (±16.1)	<2 × 10^−16^
Emotional well-being	6.4 (±1.9)	7.9 (±1.6)	2.7 × 10^−^^13^
Physical well-being	6.1 (±1.8)	7.8 (±1.6)	2.7 × 10^−^^14^
Systolic BP, mmHg	131.9 (±19.3)	119.7 (±15.8)	1.3 × 10^−^^11^
Diastolic BP, mmHg	83.8 (±10.4)	76.9 (±9.6)	1.1 × 10^−^^10^
Glucose, mmol/L	5.20 (±0.86)	4.46 (±0.93)	3.54 × 10^−^^10^
Triglycerides, mmol/L	1.45 (±0.66)	1.07 (±0.31)	8.34 × 10^−^^9^
Cholesterol, mmol/L	5.77 (±1.16)	5.09 (±1.16)	<2 × 10^−^^16^
AP, µkat/L	1.16 (±0.33)	1.12 (±0.32)	0.0025
GPT, µkat/L	0.54 (±0.35)	0.71 (±0.45)	1.71 × 10^−08^
GOT, µkat/L	0.34 (±0.14)	0.54 (±0.26)	<2 × 10^−16^
GGT, µkat/L	0.63 (±0.68)	0.44 (±0.38)	2.41 × 10^−8^
GFR, mL/min/1.73 m^2^	92.78 (±13.68)	90.16 (±14.32)	6.32 × 10^−5^
Creatinine, µmol/L	69.09 (±13.47)	71.69 (±14.19)	1.93 × 10^−5^
Urea, mmol/L	4.73 (±1.22)	3.30 (±1.27)	<2 × 10^−16^
Uric acid, µmol/L	358.9 (±83.64)	533.6 (±133.6)	<2 × 10^−16^
GSH, µmol/g Hb	4.63 (±1.3)	4.50 (±1.2)	0.27
Catalase, U/mg Hb	194.2 (±33.6)	192.6 (±36.0)	0.52
TAC, mmol DPPH/L	0.88 (±0.1)	1.02 (±0.1)	<2 × 10^−16^
TBARS, µmol/L	6.5 (±1.3)	6.0 (±1.1)	5.9 × 10^−13^
GR, U/g Hb	8.3 (±1.8)	8.3 (±2.1)	0.87
GPx, U/g Hb	24.1 (±3.9)	24.0 (±4.1)	0.72
